# Treatment of Hyperparathyroidism (SHPT)

**DOI:** 10.1590/2175-8239-JBN-2021-S107

**Published:** 2021-12-03

**Authors:** Fabiana Rodrigues Hernandes, Patrícia Goldenstein, Melani Ribeiro Custódio

**Affiliations:** 1 Faculdade Santa Marcelina, São Paulo, SP, Brazil.; 2 Hospital Israelita Albert Einstein, São Paulo, SP, Brazil; 3 Universidade de São Paulo, Faculdade de Medicina, São Paulo, SP, Brazil.

## Patients with CKD G3-5

Optimal PTH levels are not established for patients with CKD G3-5 (Evidence)

1.1 If serum PTH levels are progressively increasing or persistently above the reference value for the method, serum levels of calcium (Ca), phosphorus (P), urinary fractional excretion of P (if available), alkaline phosphatase (AP) and 25-hydroxyvitamin D (25-vit D) should be evaluated (Evidence). 

1.2 The changes found should be corrected: Ca salts for hypocalcemia, dietary guidance and/or use of P binders for hyperphosphatemia and use of ergocalciferol (vitamin D2) or cholecalciferol (vitamin D3) for hypovitaminosis D (Evidence).

1.3 For patients with persistently elevated PTH levels, despite correction of the parameters mentioned in sub-item 1.2, treatment with calcitriol, at an initial dose of 0.25-0.5 mcg/day, should be considered (Evidence).

## Patients with CKD G5D

PTH levels for patients with CKD G5D should be maintained within the target level of 2 to 9 times the upper value of the method.

2.1 For patients with PTH progressively elevated, even if within the target level, or above 9 times the reference value for the method, Ca and P control measures and the use of vitamin D analogues (such as paricalcitol) and/or calcimimetics (such as cinacalcet) should be implemented (Evidence).

When choosing the drug to start treatment of SHPT, serum levels of Ca and P should be considered.

3.1 In the presence of hypercalcemia and/or hyperphosphatemia, the use of paricalcitol should be avoided. In this case, it is recommended to start treatment with cinacalcet (Evidence).

3.2 In case of hypocalcemia, the use of cinacalcet should be avoided until hypocalcemia is corrected. It is recommended to start treatment with paricalcitol (Evidence).

3.3 The use of oral calcitriol as pulse therapy is reserved for patients on conservative treatment, on peritoneal dialysis, and those with persistent post-Tx SHPT with graft dysfunction (Opinion).

Patients treated with paricalcitol who develop hypercalcemia and/or hyperphosphatemia should have the medication dose reduced or suspended and, if necessary, cinacalcet added to the therapeutic regimen (Evidence). 

Patients treated with cinacalcet, who develop hypocalcemia, should have their dose reduced or suspended and, if necessary, paricalcitol added to the therapeutic scheme (Evidence).

The association of paricalcitol and cinacalcet is recommended to optimize control of serum PTH levels (Evidence).

Titration of paricalcitol and cinacalcet doses could be done every 2-4 weeks, with monitoring of serum Ca, P, and PTH levels, in order to keep them within their target levels (Opinion).

Patients with serum PTH levels below 150 pg/mL should have the use of paricalcitol and/or cinacalcet suspended (Evidence).

Patients who do not respond to pharmacological treatment of SHPT, with paricalcitol and/or cinacalcet, should be referred for parathyroidectomy (Evidence).

## Rational

SHPT is a progressive disease, associated with increased morbidity and mortality. Despite the restrictions, only biochemical changes guide therapeutic intervention. In addition, therapies for the control of SHPT have several limitations, making it difficult to achieve the current goals set by KDIGO^1^. 

The optimal serum PTH levels for patients with CKD G3-5D have not yet been established. In the early stages of CKD, in response to the loss of kidney function, PTH elevation occurs, aiming to maintain Ca and P levels within normal ranges. It is not always possible to distinguish when this change in PTH becomes abnormal, so when there is a progressive elevation, a more frequent dosing of this hormone should be performed. Serum AP levels, analyzed in conjunction with PTH, are important for following the progression and treatment of SHPT, at all stages of CKD^2^.

For patients on conservative treatment, Ca supplementation should be done only in those with hypocalcemia, to avoid the risk of vascular calcification^3^. In the presence of hyperphosphatemia, it is recommended starting a low P diet and Ca-based binders, monitoring fractional excretion of P^4^.

Hypovitaminosis D is associated with the progression and severity of SHPT, and its treatment with vitamin D supplementation is recommended^5^ ^-^ ^7^. It is important to remember that calcitriol should not be used in this situation, just as presentations of vitamin D, associated with Ca or other vitamins, are not recommended.

The use of vitamin D analogue, oral calcitriol, in patients with CKD 3-5D has been discussed. KDIGO 2009 suggests that moderate elevations in PTH are likely to correspond to an adaptive response to loss of kidney function, as previously discussed, with the use of calcitriol reserved for patients with progressive elevation of PTH^8^ ^,^ ^9^.

As with conservative treatment, the optimal serum PTH levels for dialysis patients are not established. Factors related to the methodology employed in PTH measurement, the lack of correlation between bone histology and intermediate PTH values, make it difficult to determine optimal levels of this hormone^10^ ^,^ ^11^. It is known that the extremes of PTH values should be avoided, since there are studies showing increased mortality associated with excessively low or increased levels of PTH^12^ ^,^ ^13^. Thus, KDIGO suggested values between 2 and 9 times the upper value of the method, and if there is a trend of increasing PTH serum levels, a therapeutic scheme should be proposed, aiming at returning to the suggested levels^1^.

With the progression of SHPT, there is a histological modification of the parathyroid glands. Diffuse hyperplasia becomes nodular, with decreased expression of vitamin D receptors (VDR) and Ca-sensing receptor (CaR), which may result in resistance to pharmacological treatment. Thus, besides controlling serum levels of Ca, P, and vitamin D, drugs that act on the modulation and/or expression of these receptors are more effective in the treatment of SHPT, such as vitamin D analogues (paricalcitol) and calcimimetics (cinacalcet)^14^ ^,^ ^15^. All of these are equally considered first-line drugs to start the treatment of SHPT. Thus, the criterion for drug choice should be based on serum levels of Ca and P. If their serum levels are within the normal range, either medication, i.e. paricalcitol or cinacalcet, could be started at the discretion of the nephrologist. In the presence of hyperphosphatemia and/or hypercalcemia, treatment should be initiated with cinacalcet. On the other hand, if the patient has hypocalcemia, treatment should be with paricalcitol.

Calcitriol, a nonselective VDR activator, was the first therapeutic agent introduced for the treatment of SHPT. Oral calcitriol could be administered as intermittent high dose (pulse therapy) in patients on conservative treatment, on peritoneal dialysis and in those with persistent post-Tx HPT with graft dysfunction. Treatment with calcitriol may lead to unwanted side effects, such as hypercalcemia and/or hyperphosphatemia due to greater intestinal absorption of Ca and P, increasing the risk of vascular calcification and mortality^14^. Another complication of the use of calcitriol is excessive suppression of PTH, which favors the development of adynamic bone disease, reiterating the need to monitor treatment with PTH and AP measurements^16^.

Paricalcitol, a selective VDR activator, is a drug with a mechanism of action that differs from calcitriol in that it has a greater affinity for the parathyroid gland than for the intestine. Thus, it promotes PTH suppression with a lower incidence of side effects, such as hyperphosphatemia and/or hypercalcemia. Its efficacy and tolerance are well described^17^, although in our context only presentation IV is available. Comparative studies between calcitriol and paricalcitol have shown that paricalcitol suppresses PTH faster and with lower occurrence of hypercalcemia^18^, and that patients in use of paricalcitol seem to have longer survival^19^. Another study demonstrated conflicting results, for example, not evidencing the superiority of paricalcitol over calcitriol in reducing serum PTH levels or in the onset of hypercalcemia and/or hyperphosphatemia^20^.

Several factors are associated with the lack of response to the treatment with calcitriol/paricalcitol, including the development of monoclonal hyperplasia of the (autonomous) parathyroid glands, which have reduced number of VDR and CaR, in addition to the development of hypercalcemia and/or hyperphosphatemia, which requires dose reduction or drug discontinuation^14^ ^,^ ^21^.

Cinacalcet, an oral calcimimetic, allosterically modulates CaR, increasing the sensitivity of parathyroid cells to extracellular Ca, decreasing PTH production and secretion and the concentration of serum Ca and P^22^ ^,^ ^23^. It is indicated in the treatment of SHPT as the drug of first choice, even with serum levels of calcium and phosphorus within normal ranges, or in the absence of patient's response to the treatment with calcitriol/paricalcitol, due to hyperphosphatemia and/or hypercalcemia^1^. The choice between cinacalcet and paricalcitol as monotherapy is not always easy, due to the particularities of each drug, even when Ca and P levels allow it^24^. Studies report that both drugs are equally effective in controlling mild or moderate SHPT in conjunction with the other standard measures^25^ ^,^ ^26^. Besides its classical actions, studies have shown other beneficial effects of cinacalcet, such as reduced progression of vascular calcification, reduction in cardiovascular events^27^ ^-^ ^29^ and incidence of clinical fractures^30^.

The prescription of cinacalcet should always start at the lowest dose (30 mg) and, if necessary, be increased every 2-4 weeks, with new controls of Ca, P and PTH. It is worth noting that, for better absorption of the drug, cinacalcet should always be administered immediately after the major meals of the day. The most common side effects of cinacalcet are hypocalcemia and gastrointestinal intolerance^14^. The management in case of hypocalcemia depends on the severity, but it generally requires lowering the dose of cinacalcet, association of vitamin D analogues and, if necessary, Ca supplementation^31^, which, if recommended, may also be done with Ca-based P binders. In cases of severe hypocalcemia, suspension of cinacalcet is recommended until Ca values are within normal range. With respect to gastric intolerance, which generally occurs after doses equal to or greater than 60 mg, we recommend, before discontinuing cinacalcet, fractioning the doses according to the patient's preference. The association of proton pump inhibitors and/or antiemetics often improves gastric intolerance to cinacalcet^32^.

Treatment failure with cinacalcet is mainly related to its side effects and to the presence of severe SHPT^33^. Thus, considering also the factors that lead to calcitriol/paricalcitol inefficiency, a strategy to be considered is the association of the two drugs, especially for patients with severe SHPT. It is worth noting that these drugs, besides presenting different mechanisms of action, have complementary action, since cinacalcet, by activating CaR, also increases the expression of VDR^34^. Another advantage of this association is that cinacalcet minimizes the side effects of calcitriol/paricalcitol and vice versa. The study by Moe et al. showed that the addition of cinacalcet to the treatment of a group of patients who were already using calcitriol, its analogues or selective VDR activators, allowed a greater proportion of patients to achieve adequate levels of Ca, P and PTH^35^. 

The use of cinacalcet in conservative treatment is off label and controversial among nephrologists^36^ ^,^ ^37^, but it has been considered in cases of patients who, despite the use of 25 vitamin D and calcitriol, persist with elevated PTH. However, in these cases, the association of cinacalcet is performed in low doses, 30 mg, and may be used daily, on alternate days or 3 times a week, according to the patient's needs. The use of cinacalcet in these conditions requires more frequent monitoring of Ca, P, and PTH^38^ ^,^ ^39^.

SHPT is a disease with complex pathophysiology, and an effective treatment is likely to include several drugs. And for patients who do not respond to clinical therapy, that is, who develop refractory SHPT, parathyroidectomy is indicated^40^ ^,^ ^41^.
